# Novel In Silico mRNA vaccine design exploiting proteins of *M. tuberculosis* that modulates host immune responses by inducing epigenetic modifications

**DOI:** 10.1038/s41598-022-08506-4

**Published:** 2022-03-17

**Authors:** H. Al Tbeishat

**Affiliations:** Al-Ghadaq Pharmaceutical Company, Amman, 11934 Jordan

**Keywords:** Computational biology and bioinformatics, Drug discovery, Immunology

## Abstract

*Tuberculosis* is an airborne infectious disease caused by *Mycobacterium tuberculosis*. BCG is the only approved vaccine. However, it has limited global efficacy. Pathogens could affect the transcription of host genes, especially the ones related to the immune system, by inducing epigenetic modifications. Many proteins of *M. tuberculosis* were found to affect the host’s epigenome. Nine proteins were exploited in this study to predict epitopes to develop an mRNA vaccine against tuberculosis. Many immunoinformatics tools were employed to construct this vaccine to elicit cellular and humoral immunity. We performed molecular docking between selected epitopes and their corresponding MHC alleles. Thirty epitopes, an adjuvant TLR4 agonist RpfE, constructs for subcellular trafficking, secretion booster, and specific linkers were combined to develop the vaccine. This proposed construct was tested to cover 99.38% of the population. Moreover, it was tested to be effective and safe. An in silico immune simulation of the vaccine was also performed to validate our hypothesis. It also underwent codon optimization to ensure mRNA’s efficient translation once it reaches the cytosol of a human host. Furthermore, secondary and tertiary structures of the vaccine peptide were predicted and docked against TLR-4 and TLR-3.Molecular dynamics simulation was performed to validate the stability of the binding complex. It was found that this proposed construction can be a promising vaccine against tuberculosis. Hence, our proposed construct is ready for wet-lab experiments to approve its efficacy.

## Introduction

*Tuberculosis* is an infectious disease caused by the pathogen *Mycobacterium tuberculosis*. In the World Health Organization (WHO) 2020 report, approximately 10 million people were diagnosed with TB and 1.2 million deaths during 2019^1^. Primarily, *M. tuberculosis* infects macrophages. However, they also have neutrophils and dendritic cells as hosts. Once *M. tuberculosis* manages to enter the host, they may stay still for a long time or get removed by the immune system. It depends on two factors; the strain virulence of Mycobacteria and the milieu of the infected macrophages. Many phagocytic vacuoles get fused with the lysosome. Hence the bacterial molecules reside in the formed phagolysosome. Proteolysis of pathogens by phagolysosomes can output peptides that can be antigenic or immunogenic^2^.

There are many available first and second lines of treatments for TB^3^. However, there is a challenge of multidrug resistance to TB drugs^4^. The only approved TB vaccine is Bacillus Calmette–Guérin (BCG). The route of injection is intradermal. However, this vaccine is limited in use due to its variability in efficacy worldwide^5,6^. Research on vaccine development was advanced through the years. The conventional approaches can successfully induce immunogenicity and stocking durable protection. Examples include living attenuated and inactivated bacteria and subunit vaccines^7^. However, many novel methods such as peptide-based vaccines and DNA vaccines showed promising opportunities for developing scalable and rapid vaccines. Nevertheless, peptide-based vaccines showed a low immunogenicity index^8^, while pDNA showed a risk of insertional mutagenesis when used inside DNA vaccines^9^. However, mRNA vaccines were found to be more efficacious than DNA vaccines as it was found that mRNA vaccines can potentially address these concerns of safety and efficacy. It is believed that mRNA therapeutics are non-infectious entities because of their lack of genomic integration and replication except for rare cases of recombination between single-stranded RNA molecules^10^. There is no need for mRNA sequences to pass through the nuclear envelope. Therefore translation for the sequences happens outside the nucleus^11^. Moreover, it is considered highly safe because of the natural degradation and adjustable half-life of mRNA vaccines^12^. These kinds of vaccines also displayed quick and low-cost production^13^. Immunoinformatics is a growing branch of bioinformatics. It is concerned with the use of computational analysis of immunological data. This field also utilizes computational tools to design *in silico* vaccines. These tools predict potential antigens and epitopes that could be harnessed in a vaccine. Accordingly, immunoinformatics can minimize the cost and timeframe of vaccine development^14^.

Microorganisms use important regulators that act as protective measures by affecting gene function without any difference in the DNA sequence called epigenetics. Many infectious agents induce various epigenetic modifications that affect the transcription of genes in the host, especially the immune cells^15^. They induce these modifications by their several by-products and metabolites. These modifications include RNA-based modifications, histone regulation, chromatin remodeling, DNA methylation, and hydroxymethylation. In TB, epigenetic strategies study their role during the latent and active TB diseases. These products are vital for TB disease as they affect the host genes of the innate and adaptive immune systems. This study investigates nine proteins among these products. Histone Acetyltransferase Rv3423.1 (accession number-P9WKY5) is a secreted mycobacterial (H37Rv strain) protein that affects the expression of anti-inflammatory genes of the host^16^. Moreover, the DNA methyltransferase Rv2966c (accession number-I6XFS7) of the M. tuberculosis affects histones H3 and H4 and thus transcription in the host^17^. M. tuberculosis uses the acetyltransferase EIS (Rv2416c accession number-P9WFK7) to stimulate the overexpression of human IL-10. It does so by inducing the acetylation of H3 of the gene promoter. This protein is also involved in the evasion of autophagy and persistence intracellularly^18^. As a defense mechanism in TB infections, the esxL (Rv1198 accession number-P9WNJ5) induces H3K9me2/3 and, thus, suppressing the expression of MHC-II and CIITA in infected macrophages^19^. The cobB (Rv1151c accession number-P9WGG3) in *M. tuberculosis*is a Sir2 like protein that modifies the compact structure of the DNA. It specifically induces deacetylation of acetylated HU^20^. A 6 kDa early secretory antigenic target (esxA) is a secreted protein that modulates the immune response to infection and *M.tuberculosis* fleeing into the cytoplasm of the host. It is also a potent human T-cell antigen^21,22^. *M. tuberculosis* possesses proteins with methylase activities (e.g. the probable DNA methylase (Modification methylase) (Rv3263 accession number-P96868)^17^ and spoU (Rv3366 accession number-O50394)^17^ as proteins with methylase activity. In the headmost streak of protection against TB infections, the mycobacteria use the secreted methyltransferase erm(37) (Rv1988accession number-Q10838) that modulates methylation of H3 and suppresses involved genes of the host^23^.

The underlying mechanisms that *M. tuberculosis* effectively uses in TB infection are varied, but few are fully disclosed. The effect of methylation could either lead to activation or suppression of gene expression if it occurs respectively on lysine or arginine. However, if tri-methylation of H3K9occurs, gene expression is suppressed. This DNA methylation of immune genes refers to “trained immunity”^24^. This theory supports the memory of innate immunity related to the fast and robust response to secondary exposure to infection^25^. Thus, a premature revelation of TB liable persons is possible in different populations as these epigenetic effects are heritable. *M. tuberculosis* proteins with histone modification are Rv1198, Rv1988, and Rv2966c, which induce histone methylation during TB infection. The Rv2966c resembles mammalian Dnmt3L DNA methyltransferase (DNMTs), and it can interact with H3and H4^17^.

In this study, we aimed at designing a novel multi-epitope mRNA vaccine by exploiting the proteins related to *M. tuberculosis* that have roles in the modification of the epigenome of the host. The used approach of engineering this vaccine construct is purely *in silico*. We used many computational tools to predict B cell, helper T lymphocytes (HTL), and cytotoxic T lymphocytes (CTL) epitopes out of the included protein sequences. These epitopes were assessed to be potent antigenic, non-allergenic and non-toxic. These peptides were also assessed to see if they could develop any autoimmunity. Moreover, As an adjuvant, the TLR4 agonist (RpfE) peptide was inserted to improve the immunogenicity of the vaccine construct. We also performed molecular docking between T lymphocyte epitopes and MHC alleles to prove the concept. Subsequently, we assessed the vaccine construct’s population coverage, antigenicity, allergenicity, toxicity, various physicochemical properties, and *in silico* immune simulation to validate our hypothesis. Furthermore, the mRNA vaccine was codon optimized to ensure its proper translation inside the host. Moreover, the secondary and tertiary structures were predicted for the peptide sequence of the vaccine. Ultimately, the resulting 3D structure was docked against TLR-4 and TLR-3. Then, we validated the stability of the formed complex using molecular dynamics simulation.

## Results

### Prediction and estimation of B-cell epitopes

We only selected the top five epitopes predicted from the ABCpred webserver from each included protein. In addition, we filtered only the epitopes that were antigenic, non-allergenic, and non-toxic to be included further in the vaccine construct. We used VaxiJen, AllerTop, and ToxinPred web servers, respectively. Moreover, we screened all predicted epitopes to see if they have homologs among Homo Sapiens (Taxid:9606) to exclude from the vaccine construct, which could induce autoimmunity. Therefore, we excluded all peptides with an E value less than 0.05. Altogether, we checked also that all predicted epitopes lie in the investigated proteins’ conserved regions. All variants of selected proteins were downloaded from the NCBI database and aligned in the Bioedit 7.2 program. In total, we settled on eight B-cell epitopes extracted from the nine studied proteins to include in the vaccine construct (Table 1).

**Table 1:** List of candidate epitopes for vaccine design.

Cell Type	Sequence of Epitope
CD8 + Cytotoxic T Lymphocytes	CPIAPGRGA
	TGLAVLDLY
	DLVLADPPY
	NMAQTDSAV
	YEQANAHGQ
	IRESASQAL
	EVNPEPTPL
	MTEQQWNFA
	WGGSGSEAY
	GELADVDVGI RLLFVSPRI
	SLNLSNAAAV
	FTLTVGLML GLVAADLVL
	IRLPGRPFRV
	HLGYKCSIRK
	ARNIEALGL
CD4 + helper T Lymphocytes	TEQYSGLCPIAPGRG
	WPQRVYGDTRLELAE
	RNFQVIYEQANAHGQ
	CRAAWLTLRDRRTKR
	DLFMLRQIHFAPRLT
B Lymphocyte	SGLCPIAPGRGAGLQP
	LGLSGATLRRGAVAAV
	ASEGGIYGRFGYGPAT
	GQKVQAAGNNMAQTDS
	PDLALARGTAVIEVNP
	KWDATATELNNALQNL
	DGVAGNPPYIRFGNWA
	ATLADTHITGQVRIPM

### Prediction and estimation of CTL epitopes

We predicted possible CTL epitopes from the nine proteins selected for this study using the IEDB database. We chose only epitopes with IC50 over 500 for further study. Additionally, we only further selected the epitopes that were antigenic, non-allergenic, non-toxic, and non-homologs. Finally, we selected 17 epitopes to include in the vaccine construct that lie in the conserved regions of the proteins (Table 1).

### Prediction and estimation of HTL epitopes

We selected several possible HTL epitopes from studying the nine proteins mentioned above of *M. tuberculosis*. Only the antigenic and non-allergenic, non-toxic, and non-homologs were investigated for their possibility to induce Il-4, Il-10, and IFN-*γ*. Finally, we included five possible epitopes that exist in the conserved regions of the proteins and induced the cytokines mentioned above in the vaccine (Table 1).

### Molecular Docking between MHC alleles and selected T lymphocyte epitopes

**Figure 1:**
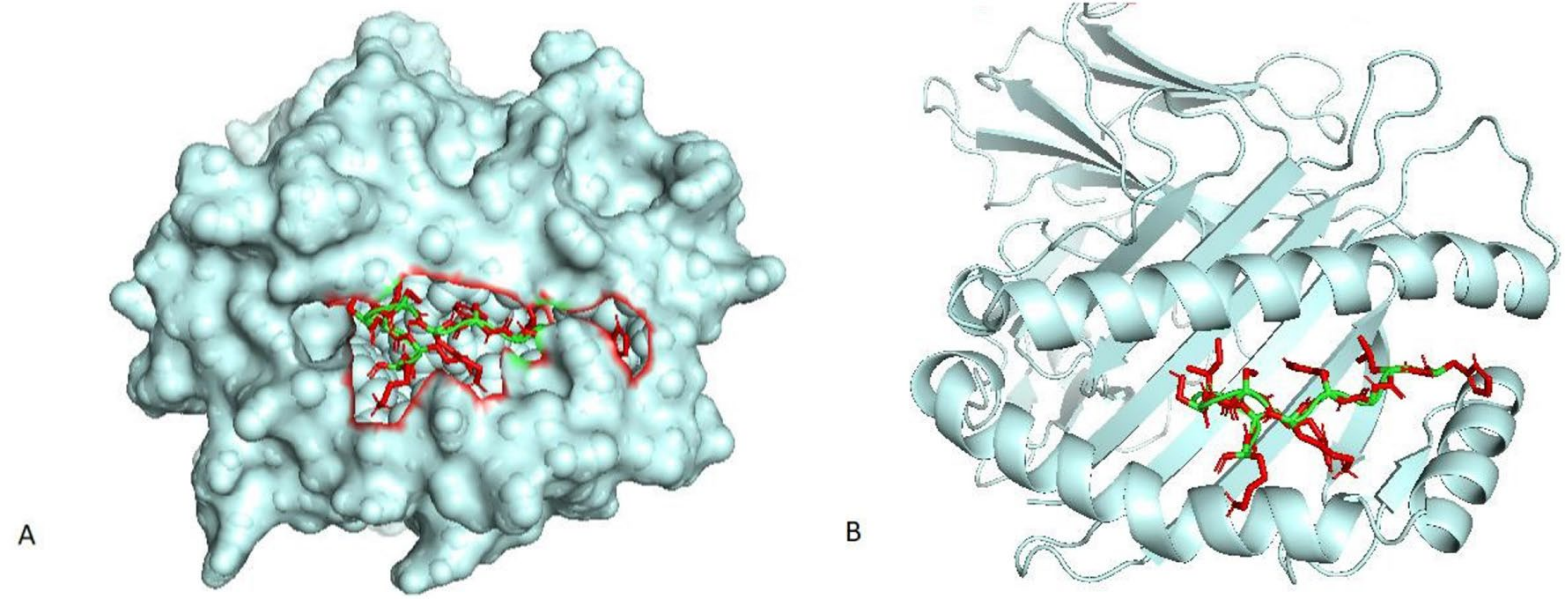
Visualization of the docking between the epitope HLGYKCSIRK and its corresponding MHC allele (HLA-A*03:01) using the PyMol software: (**A**) Surface View. (**B**) Cartoon View.

**Figure 2:**
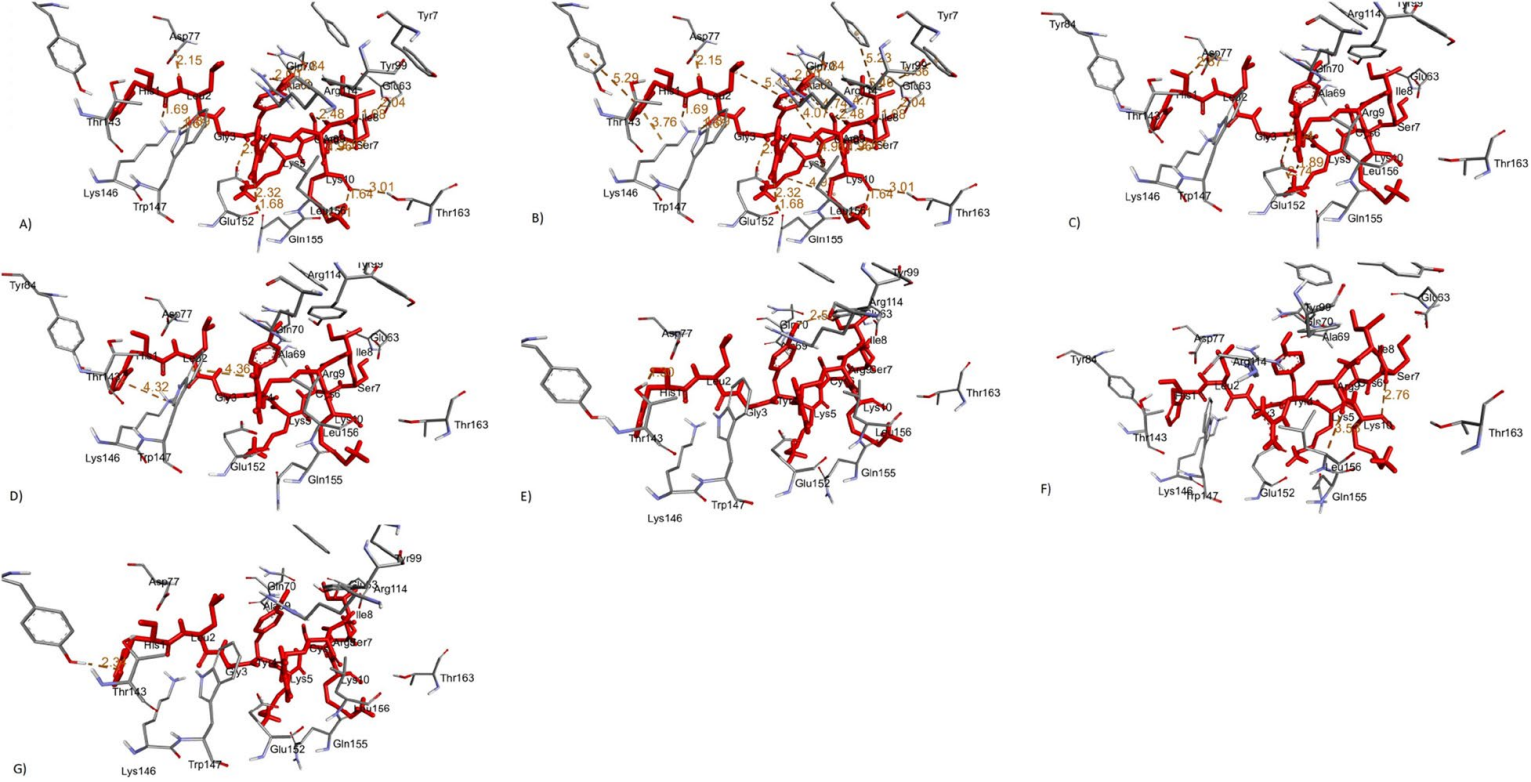
Different Interactions between the epitope and its corresponding MHC allele visualized using the discovery studio. (**A**) Conventional Hydrogen Bonds (**B**) Hydrophobic Interactions (**C**) Salt Bridge, attractive Charge interactions (**D**) Cation-Pi interactions (**E**) Donor-Donor Clash (**F**) Carbon Hydrogen Bond (**G**) Pi Donor Hydrogen Bond.

The 22 lymphocyte epitopes recognized a total of 65 MHC alleles. Some of these epitopes recognize one allele while others can bind to multiple alleles, with one epitope can recognize 32 alleles (Table 2). Out of these epitopes, we performed molecular docking for six epitopes and their parallel MHC alleles (Table 3). We downloaded all the crystallographic structures of chosen MHC alleles from the RCSB PDB. Please refer to (Table 3) for their PDB IDs. However, the molecular docking results obtained from ClusPro 2.0 in terms of binding affinity are in Table 4. The epitope HLGYKCSIRK displayed the strongest binding affinity with its corresponding MHC allele (HLA-A*03:01) with a − 8.502 Kcal/mol value. Subsequently, we found that this epitope skillfully fit inside the binding cleft of its corresponding allele (Fig. 1A,B). Later on, we evaluated all possible interactions between the epitope and the various residues of the MHC allele. We found seven types. The type, numbers of interactions, and length of the bonds in angstrom are in Fig. 2 and Table 5.

**Table 2:** Selected T lymphocyte epitopes (CTL + HTL epitopes) and their corresponding MHC alleles.

Protein No	CTL Epitopes	MHC I binding Alleles	HTL Epitopes	MHC II binding Alleles
1	CPIAPGRGA (21)	HLA-B*07:02	TEQYSGLCPIAPGRG (14)	HLA-DRB1*10:01, HLA-DRB1*01:01, HLA-DRB1*04:01, HLA-DRB1*04:05, HLA-DQA1*02:01/DQB1*03:01, HLA-DQA1*05:01/DQB1*03:02, HLA-DQA1*05:01/DQB1*03:03, HLA-DQA1*02:01/DQB1*03:03, HLADQA1*03:01/DQB1*03:01, HLA-DQA1*05:01/DQB1*04:02
2	TGLAVLDLY (43) DLVLADPPY (114)	HLA-A*30:02 HLA-B*35:01, HLA-A*29:02, HLA-B*15:02	WPQRVYGDTRLELAE (168)	HLA-DRB1*03:01, HLA-DPA1*03:01/DPB1*04:02, HLA-DPA1*02:01/DPB1*01:01, HLA-DPA1*01:03/DPB1*02:01
	ARNIEALGL (83)	HLA-B*27:05		
3	NMAQTDSAV (81)	HLA-B*18:01	RNFQVIYEQANAHGQ (59)	HLA-DRB4*01:01, HLA-DRB1*10:01, HLA-DRB1*04:01, HLA-DRB1*08:02, HLA-DQA1*01:02/DQB1*05:01, HLA-DRB1*01:01, HLA-DRB1*04:05, HLA-DRB1*16:02, HLA-DRB5*01:01, HLA-DRB3*03:01, HLA-DRB1*15:01
	YEQANAHGQ (65)	HLA-A*02:06		
4	IRESASQAL (217) EVNPEPTPL (201)	HLA-B*39:01, HLA-C*07:01 HLA-C*03:03, HLA-A*68:02, HLA-C*15:02		
5	MTEQQWNFA (1)	HLA-A*01:01, HLA-A*68:02, HLA-A*30:01		
	WGGSGSEAY (49)	HLA-B*35:01, HLA-A*29:02		
6	GELADVDVGI (298)	HLA-B*40:01, HLA-A*02:01, HLA-A*68:02	DLFMLRQIHFAPRLT (409)	HLA-DPA1*01:03/DPB1*06:01, HLA-DRB1*13:01, HLA-DRB4*01:03, HLA-DQA1*05:01/DQB1*04:02, HLADRB5*01:01, HLA-DRB1*08:01, HLA-DRB1*10:01, HLA-DRB4*01:01, HLA-DPA1*01:03/DPB1*04:01, HLA-DRB1*11:01, HLA-DRB1*01:01, HLA-DRB1*12:01, HLA-DPA1*03:01/DPB1*04:02, HLA-DRB3*03:01, HLA-DRB1*15:01, HLA-DQA1*03:03/DQB1*04:02, HLA-DRB1*04:04, HLA-DPA1*01:03/DPB1*03:01, HLA-DPA1*02:01/DPB1*01:01, HLA-DRB1*07:01, HLA-DRB1*09:01, HLA-DQA1*01:02/DQB1*05:01, HLA-DRB1*16:02, HLA-DRB1*04:01, HLA-DQA1*06:01/DQB1*04:02, HLA-DPA1*01:03/DPB1*02:01, HLADRB1*13:02, HLA-DQA1*01:02/DQB1*05:02, HLA-DRB1*04:05, HLA-DQA1*02:01/DQB1*04:02, HLADRB3*02:02, HLA-DRB1*08:02
	HLGYKCSIRK (388)	HLA-A*03:01, HLA-A*11:01	CRAAWLTLRDRRTKR (535)	HLA-DRB4*01:03, HLA-DRB1*13:01, HLA-DQA1*02:01/DQB1*04:02, HLA-DRB5*01:01, HLA-DRB1*11:01, HLA-DRB1*01:01, HLA-DRB1*08:01, HLA-DQA1*05:01/DQB1*04:02, HLA-DRB1*03:01, HLADQA1*06:01/DQB1*04:02, HLA-DRB1*16:02, HLA-DRB1*10:01, HLA-DQA1*03:03/DQB1*04:02, HLA-DRB1*04:05, HLA-DRB3*03:01
7	RLLFVSPRI (3)	HLA-A*32:01, HLA-A*02:01, HLA-A*02:06, HLA-A*30:01		
	SLNLSNAAAV (130)	HLA-A*02:01, HLA-A*02:06		
8	FTLTVGLML (142)	HLA-A*02:06, HLA-C*15:02, HLA-A*02:01, HLA-C*03:03, HLA-C*14:02		
	GLVAADLVL (118)	HLA-A*02:01, HLA-B*15:01		
	IRLPGRPFRV (87)	HLA-A*02:01, HLA-B*27:05

**Table 3:** Docking analysis of some CTL and HTL epitopes with their corresponding MHC alleles.

Type of T Lymphocyte	Epitope	MHC Alleles	PDB ID of MHC Allele
CTL	DLVLADPPY	HLA-B*35:01	4PR5
	HLGYKCSIRK	HLA-A* 03:01	3RL1
	GLVAADLVL	HLA-B* 15:01	1XR8
	IRLPGRPFRV	HLA-B* 27:05	6PYJ
HTL	TEQYSGLCPIAPGRG	HLA-DRB1*01:01	2FSE
	RNFQVIYEQANAHGQ	HLA-DRB1*15:01	1BX2

**Table 4:** Binding Affinity between the selected epitopes and their corresponding MHC alleles in (Kcal/mol).

Epitope	Allele	Binding Afinity
HLGYKCSIRK	HLA-A*03:01	− 8.502
RNFQVIYEQANAHGQ	HLA-DRB1*15:01	− 8.387
TEQYSGLCPIAPGRG	HLA-DRB1*01:01	− 8.161
IRLPGRPFRV	HLA-B*27:05	− 7.497
DLVLADPPY	HLA-B*35:01	− 7.166
GLVAADLVL	HLA-B*15:01	− 6.831

**Table 5:** The interactions involved docking the epitope HLGYKCSIRK with its corresponding MHC allele(HLA-A*03:01).

Type of Interaction	Amino Acid (Position)	Bond Length (Angstrom)
Conventional Hydrogen Bonds	ASP77	2.15 Å
	ASP77	1.69 Å
	TRP147	1.89 Å
	LYS146	1.65 Å
	GLU152	2.61 Å
	GLU152	2.32 Å
	ARG114	2.00 Å
	ARG114	1.76 Å
	GLN70	1.84 Å
	GLN155	1.68 Å
	GLN155	1.71 Å
	THR163	3.01 Å
	GLU63	2.04 Å
Hydrophobic Interactions	TYR84	5.29 Å 3.76 Å 4.74 Å
	LYS146	3.76 Å
	ALA69	4.74 Å
	PHE9	5.23 Å
	TYR99	5.46 Å
	TYR7	5.36 Å
	LEU	4.96 Å
Salt Bridge, attractive charge interactions	GLU152 ASP77	1.89 Å 2.87 Å
	GLU152	5.04 Å
	GLU152	2.74 Å
Donor-Donor Clash	THR143	1.30 Å
	TYR99	2.50 Å
Cation-Pi Interaction	LYS146	4.32 Å
	TRP147	4.36 Å
Carbon Hydrogen Bond	LEU156	3.53 Å
Pi Donor Hydrogen Bond	TYR84	2.34 Å

### Vaccine construct design

We designed the proposed vaccine construct from the N- to C- terminal, like the following: 5′ m7GCap– 5′ UTR–Kozak sequence–Signal peptide (tPA)–EAAAK linker- Adjuvant (RpfE)–GPGPG Linker–TEQYSGLCPIAPGRG–GPGPG Linker–WPQRVYGDTRLELAE–GPGPG Linker–RNFQVIYEQANAHGQ–GPGPG Linker–CRAAWLTLRDRRTKR –GPGPG Linker–GLVAADLVL–KK Linker–ATLADTHITGQVRIPM–KK Linker -DLFMLRQIHFAPRLT–KK Linker– SGLCPIAPGRGAGLQP–KK Linker–LGLSGATLRRGAVAAV KK Linker–ASEGGIYGRFGYGPAT–KK Linker–GQKVQAAGNNMAQTDS-KK Linker–PDLALARGTAVIEVNP–KK Linker–KWDATATELNNALQNL-KK Linker –DGVAGNPPYIRFGNWA–AAY linker–IRLPGRPFRV–AAY Linker–HLGYKCSIRK–AAY Linker–ARNIEALGL–AAY Linker–CPIAPGRGA–AAY Linker-TGLAVLDLY–AAY Linker–DLVLADPPY–AAY Linker–NMAQTDSAV–AAY Linker–YEQANAHGQ–AAY Linker–IRESASQAL–AAY Linker–EVNPEPTPL–AAY Linker–MTEQQWNFA–AAY Linker–WGGSGSEAY–AAY Linker–GELADVDVGI–AAY Linker–RLLFVSPRI–AAY Linker–SLNLSNAAAV–AAY Linker–FTLTVGLML–AAY Linker–MITD sequence–Stop codon–3′ UTR–Poly (A) tail.

### Evaluation of antigenicity, allergenicity, toxicity, and physicochemical properties of the vaccine construct

We tested the vaccine construct for its antigenicity, allergenicity, and toxicity using the VaxiJen, ANTIGENpro, AllerTop, and ToxinPred servers. We also calculated the physicochemical properties of the vaccine using the ProtParam server (Table 6). The vaccine stood to be antigenic, non-allergenic, and non-toxic. Moreover, all its physicochemical properties indicated that the vaccine is thermally stable. The Grand average of hydropathicity (GRAVY) was measured to be -0.300, which indicated the hydrophilic nature of the vaccine. Based on these results, this multi-epitope mRNA vaccine construct can be a potential vaccine candidate.

**Table 6:** The physicochemical properties of the translated form of the proposed mRNA vaccine.

Property	Measurement	Indication
Total Number of Amino Acids	629	Appropriete
Molecular Weight	65.574 KDa	Appropriete
Formula	C2926H4544N830O861S14	–
Theoretical pI	9.04	Basic
Total Number of Negatively Charged Residues (Asp + Glu)	49	–
Total Number of Positively Charged Residues (Arg + Lys)	59	–
Total Number of Atoms	9175	–
Instability Index (II)	33.51	Stable
Aliphatic Index (AI)	77.54	Thermostable
Grand Average of Hydropathicity (GRAVY)	-0.196	Hydrophilic
Antigenicity (Using VaxiJen)	0.8140	Antigenic
Antigenicity (Using ANTIGENpro)	0.935437	Antigenic
Allergenicity (Using AllerTop 2.0)	Non-allergenic	Non-allergenic
Toxicity (ToxinPred)	Non-toxic	Non-toxic

### Population Coverage Prediction

We measured the combined global population coverage of the 23 epitopes for their corresponding 65 alleles via the IEDB Population Coverage tool. The vaccine found that it would cover around 99.38% of the world.

### In silico immune simulation of response against the vaccine

**Figure 3:**
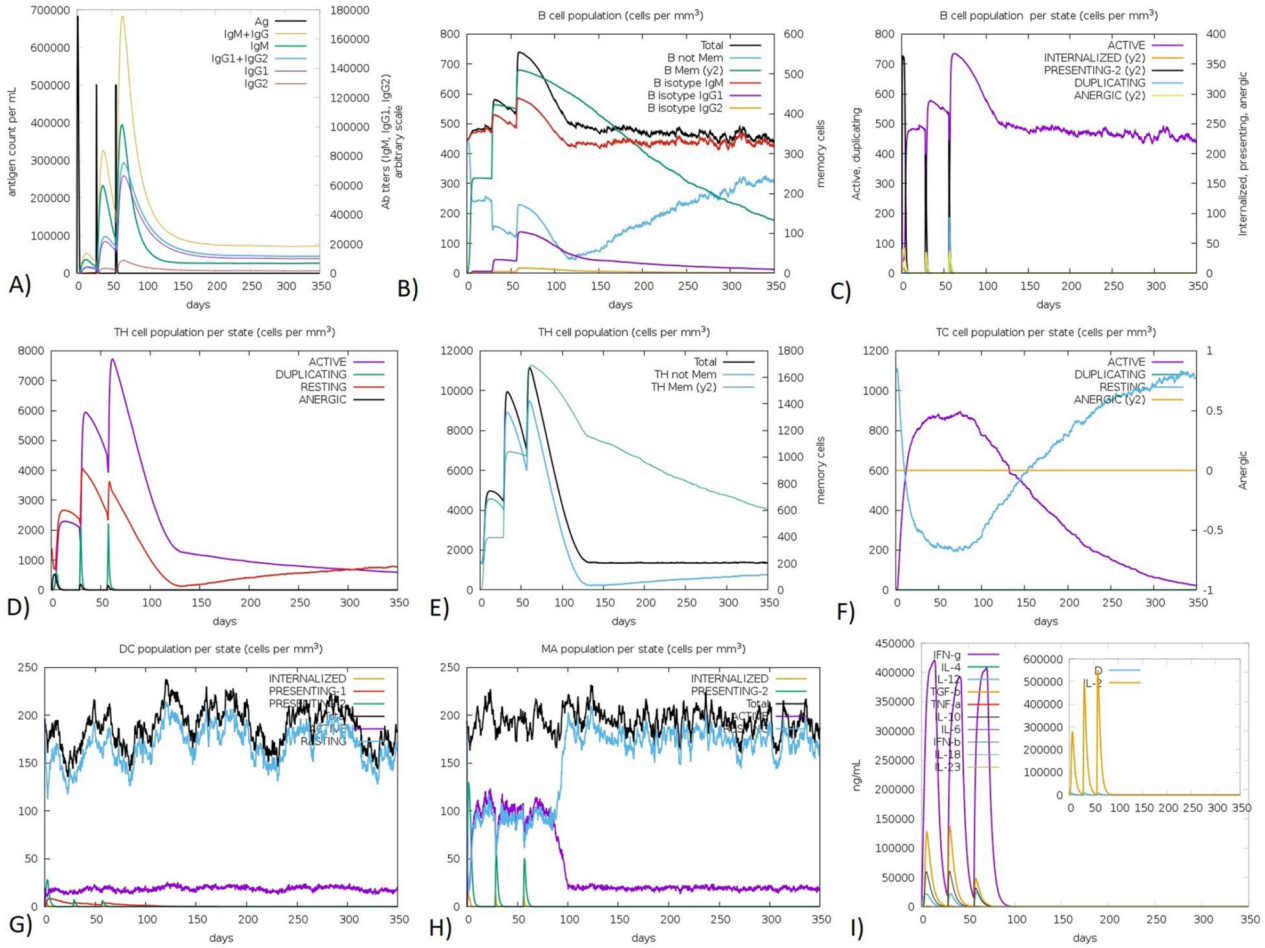
In Silico Immune Simulation against the mRNA vaccine retrieved from the C-ImmSim server. (https://kraken.iac.rm.cnr.it/C-IMMSIM/). (**A**) The immunoglobulin production after antigen injection. (**B**) The B cell population after three injections. (**C**) The B Cell Population per state (**D**) The Helper T Cell Population (**E**) The Helper T Cell Population per state (**F**) The Cytotoxic T Cell Population per state (**G**) Macrophage Population per state (**H**) Dendritic Cell Population per state (**I**) Cytokines and Interleukins Production with Simpson Index of the immune response.

We used three injections of the vaccine to simulate the immune response (Fig. 3). The second and third responses were higher than the primary ones. The produced immunoglobulin (IgM) was higher than IgG. After antigen reduction, the levels of immunoglobulins were high. This increase could indicate that immune memory could emerge from exposure to the antigen and thus indicate practiced immunity. Isotype switching and memory formation of the B cell population is evidenced due to the presence of B-cell isotypes for a prolonged duration of time. Moreover, there was an increase in CTL and HTL cells with memory generation. Furthermore, macrophage activity was enhanced, while dendritic cell activity was stable. Additionally, levels of IFN- *γ* and IL-2 were also increased. The epithelial cells were increased, which are components of innate immunity. Ultimately, the Simpson index (D) is low, which points out a difference in the immune response.

### Codon Optimization of the mRNA construct

**Figure 4:**
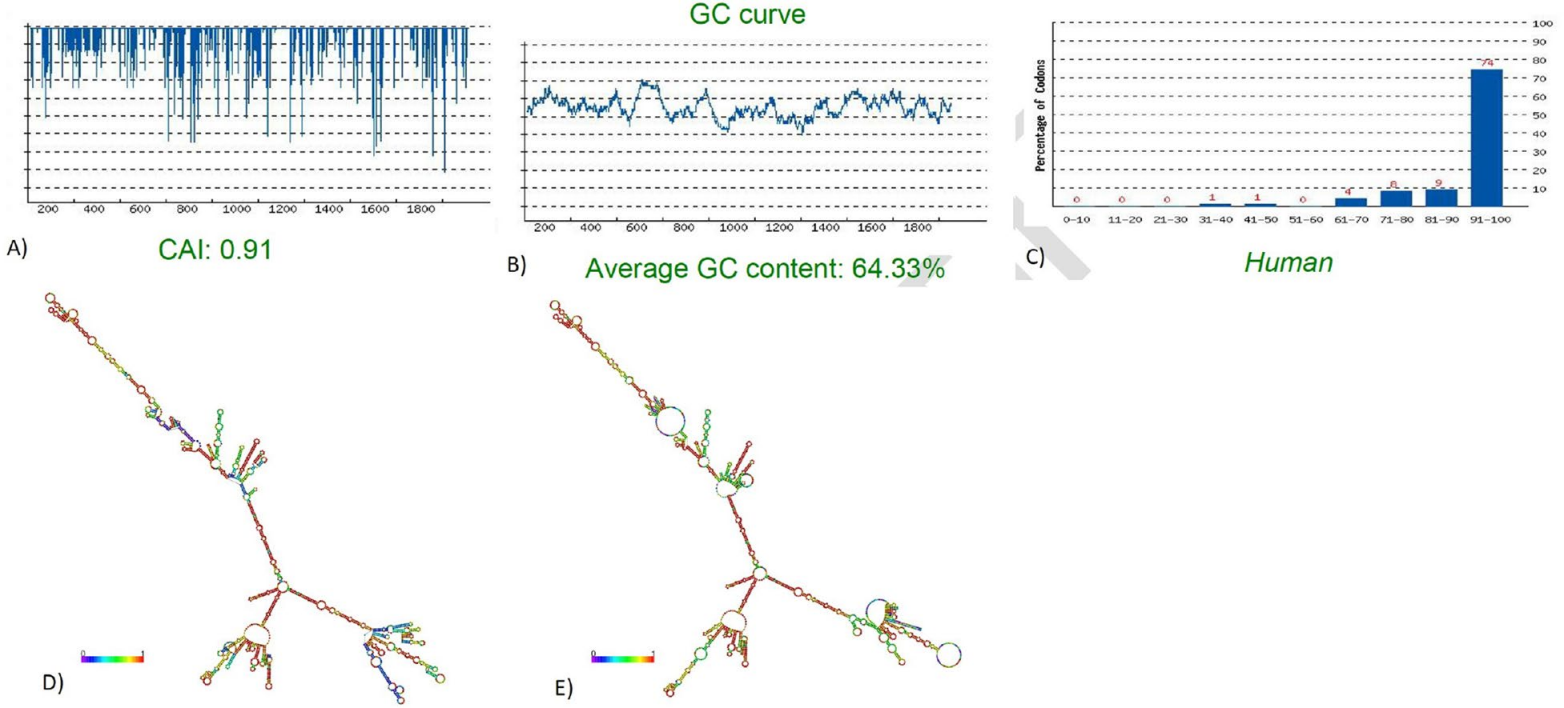
Codon optimization and mRNA vaccine structure prediction: (**A**) CAI value (**B**) GC% (**C**) CFD value (**D**) Optimal secondary structure (**E**) Centroid secondary structure of the vaccine mRNA retrieved using RNAfold Webserver (http://rna.tbi.univie.ac.at/cgi-bin/RNAWebSuite/RNAfold.cgi).

Codon optimization tools are needed to enhance the translation of the mRNA vaccine once inside host cells. Therefore, we used the GenSmart Codon Optimization tool (GS) for efficient expression in human cells. The CDS length of the mRNA was 1896 nucleotides. We used the rare codon analysis tool from GS to assess the quality of the optimized construct. The CAI value was estimated to be 0.91 (Fig. 4A). As the CAI is more significant than 0.8, it is acceptable. The optimal percentage of GC content needs to be around 30-70% to indicate that the vaccine sequence will be expressed efficiently in the human host. The average GC percentage of the optimized construct was 64.64% (Fig. 4B). The Codon Frequency Distribution (CFD) was 0% (Fig. 4C). Thus, this figure indicates that no codons could hamper the translation efficiency or function. As, any codons with a value lower than 30 could reduce or stop translational machinery.

### Prediction of the secondary structure of the mRNA

The structure of the mRNA vaccine was predicted via the RNAfold server^26^. The free energies of the structure were also assessed using this server. As an input, we used the optimized codons of the construct. We found that the mRNA will be stable when manufactured with minimum free energy (MFE) of the structure of -802.76 kcal/mol (Fig. 4D), and the secondary centroid structure of -650.48 kcal/mol (Fig. 4E).

### Prediction and validation of secondary and tertiary structures of the translated construct

**Figure 5:**
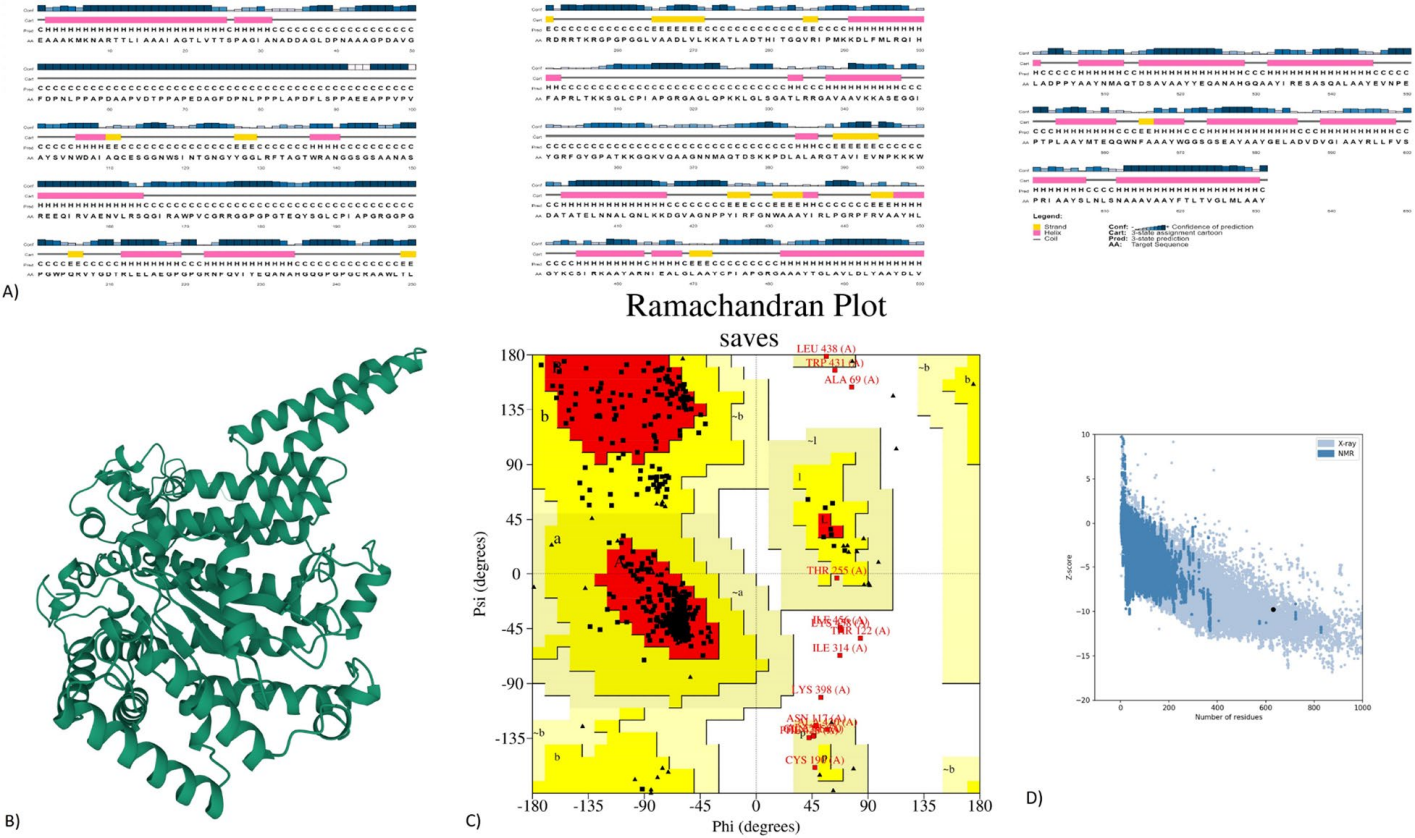
Structure prediction and validation of the peptide vaccine construct: (**A**) The secondary structure of the vaccine using the PSIPRED server (**B**) Tertiary structure of the peptide using the Robetta server (**C**) Ramachandran plot analysis using the PROCHECK server (**D**) Z-score analysis using Pro-SA webserver.

We used the PSIPRED web service to predict the secondary structure of the vaccine^27^. The alpha helices prevailed in the structure (Fig. 5A). Moreover, we predicted the tertiary structure of the peptide using the Robetta server (Fig. 5B). We then used the PROCHECK server to verify the stereochemical accuracy of the structure. This server considers both the geometry of residues and the whole structure for the prediction. In (Fig. 5C), the Ramachandran plot indicates that around 87.5% of residues were in the most favored regions, 9.6% in the additionally allowed zone, and 1.6% in the generously allowed regions. The overall quality factor was 93.5275. The ProSA-web predicted a negative Z-score (− 9.78) that indicates that the 3D protein model is very consistent (Fig. 5D).

### Conformational B-cell epitopes prediction

**Figure 6:**
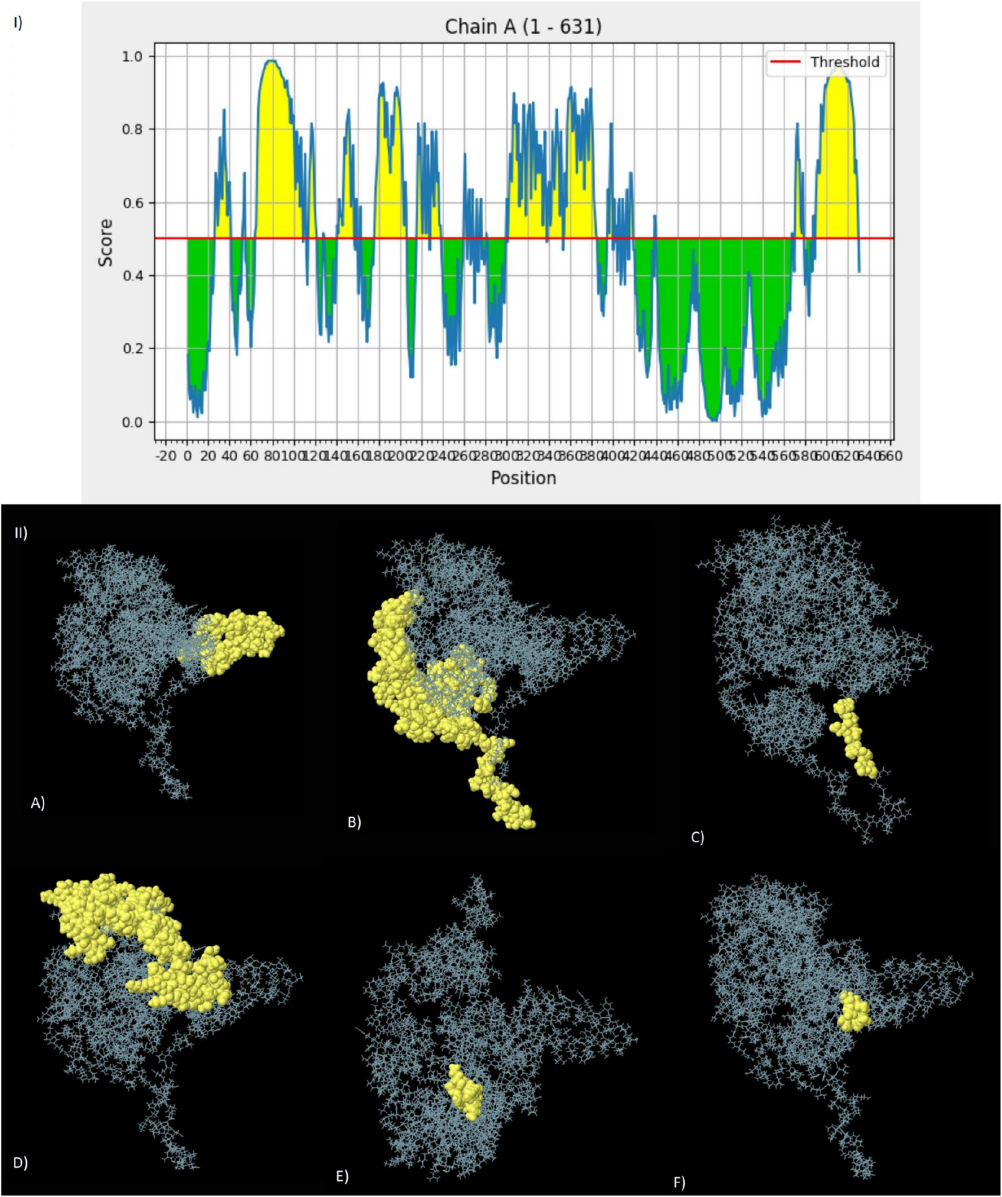
The six predicted conformational B-cell epitopes using the ElliPro tool of the IEDB database: (I) 2D diagram of the positions of conformational B-cell epitopes. (II) The 3D models of B-cell epitopes. The yellow spheres represent the conformational B-cell epitopes. (**A**) 53 residues with a score of 0.809. (**B**) 140 residues with a score of 0.726. (**C**) 10 residues with a score of 0.699. (**D**) 120 residues with a score of 0.681. (**E**) 7 residues with a score of 0.582. (**F**) 5 residues with a score of 0.515.

The folding of the model protein results in the generation of conformational B-cell epitopes. We used the ElliPro server to predict six discontinuous B-cell epitopes. A total of 335 residues were with a prediction score ranging from 0.515 to 0.809. The 2D and 3D models of the conformational B-cell epitopes are shown in (Fig. 6 I,II) respectively.

### Molecular docking of the vaccine peptide

**Figure 7:**
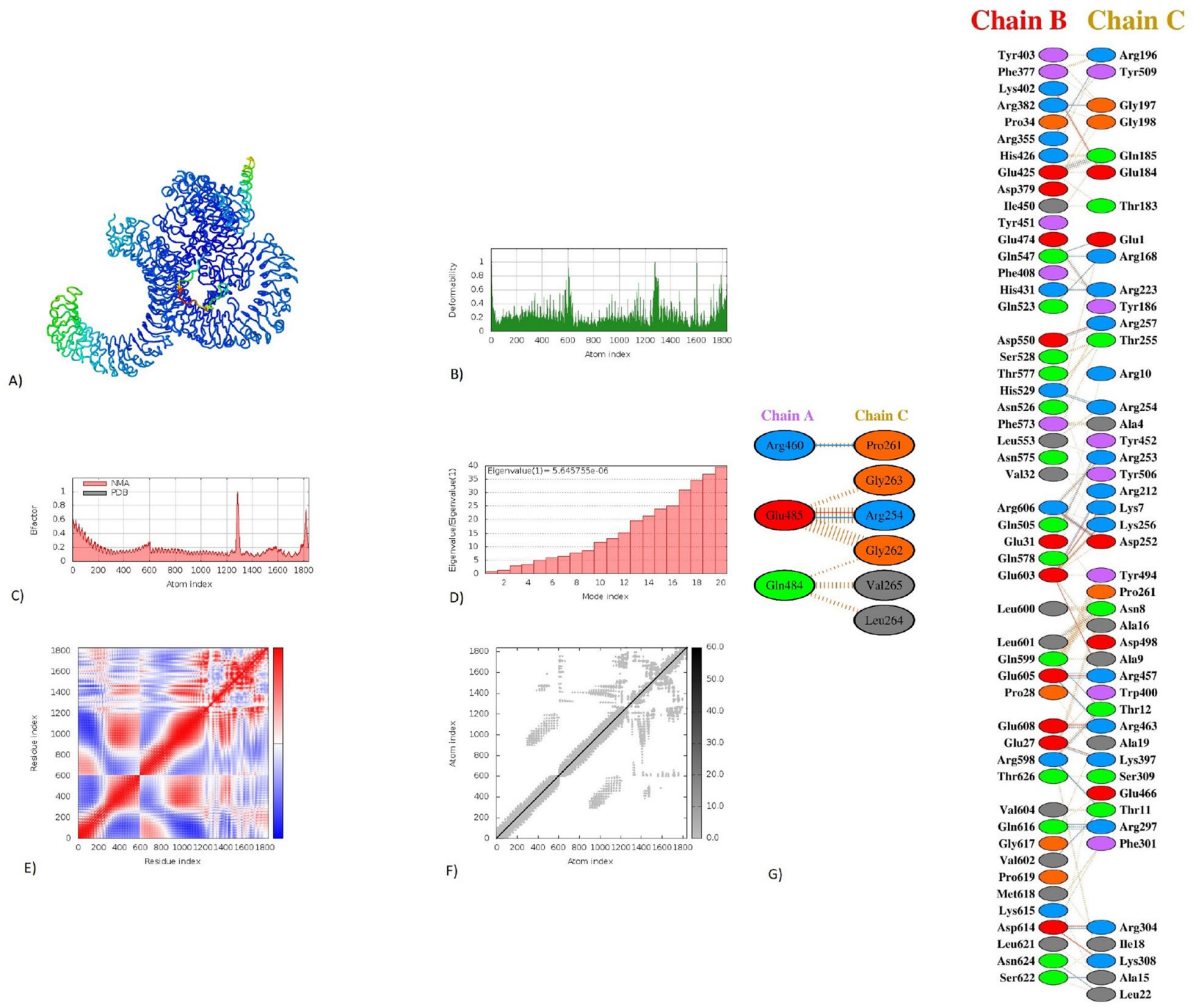
Molecular dynamics simulation, Normal Mode Analysis, and receptor-ligand interactions: (**A**) Vaccine-TLR4 docked complex using the Cluspro server (**B**) Deformability graph (**C**) B-factor graph (**D**) Eigenvalue of vaccine-TLR4 complex (**E**) Covariance matrix (**F**) Elastic network model using the iMODS server (**G**) Receptor-ligand interaction using the PDBsum webserver.

**Figure 8:**
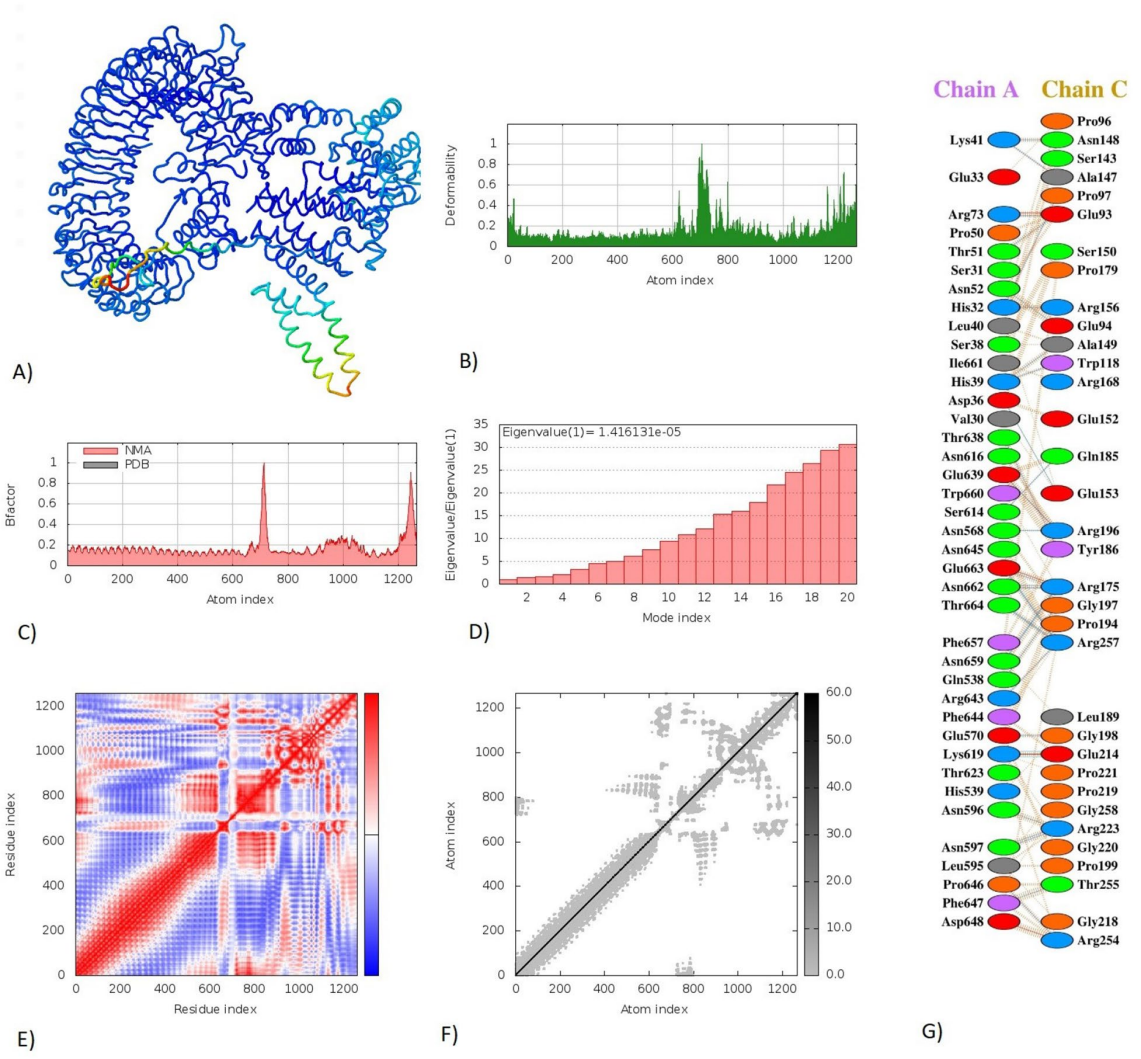
Molecular dynamics simulation, Normal Mode Analysis, and receptor-ligand interactions: (**A**) Vaccine-TLR3 docked complex using the Cluspro server (**B**) Deformability graph (**C**) B-factor graph (**D**) Eigenvalue of vaccine-TLR3 complex (**E**) Covariance matrix (**F**) Elasticnetwork model using the iMODS server (**G**) Receptor-ligand interaction using the PDBsum webserver.

Again, we used the ClusPro server to perform the molecular docking and confirm the possible interactions of the construct with the TLR-4 (Fig. 7A) and TLR-3 (Fig. 8A) receptors. Secondly, we used the PRODIGY webserver to calculate the binding affinities and dissociation constants at 37 °C for the highest cluster member for each complex separately. As a control, we used the adjuvant docked with either TLR4 or TLR3. For the TLR3-vaccine complex, the binding affinity ∆G was − 17.0 kcal mol^−1^, while the control was only − 8.1. The dissociation constant Kd (M) at 25.0 °C was for the vaccine-TLR3 complex 3.5E−13 compared to 1.1E−06 of the control. For the TLR4-vaccine complex, the binding affinity ∆G was − 13.5 kcal mol^−1^, while the control was − 9.0. The dissociation constant Kd (M) at 25.0 °C was for the vaccine-TLR3 complex 1.3E−10 compared to 2.5E−07 of the control. The specific interactions between the amino acids of the peptide vaccine (ligand) and either TLR-4 (Fig. 7G) or TLR-3 (Fig. 8G) receptors were analyzed using the PDBsum server.

### Molecular dynamics simulation

The docked vaccine-TLR3 and vaccine-TLR4 complexes were subjected to molecular dynamics simulation using the iMODS server. The receptor-ligand interaction was also assessed. The deformable loci of the construct are represented by peaks in the deformability graph (Figs. 7B, 8B). In the deformability plot, the amino acids with coiled shapes are presented. Normal mode analysis (NMA) is a computational approach for studying and characterizing protein flexibility. The relationship between the NMA and PDB areas in the uploaded complex is depicted in the B-factor graph (Figs. 7C, 8C). The eigenvalues of the docked complexes are in (Fig. 7D, 8D). To this extent, these results emphasize that the vaccine-receptor complex is with both a low deformation index, stronger stiffness, and more stability. In (Figs. 7E, 8E), the covariance matrix demonstrated the connection between amino acid duplets scattered in dynamical regions. The red color represents the correlated residues, the white represents the anti-correlated amino acid duplets, and the blue represents non-correlated residues. A connecting matrix that represents the elastic network model is employed to classify which atom pairs are connected by springs (Figs. 7F, 8F). Each chain of the complex was found to be with higher stiffness. The darker gray color indicates stiffer regions.

## Discussion

Tuberculosis is still a burden on global health. To date, the only approved anti-tuberculosis vaccine is BCG. This vaccine was established in 1921. Despite this fact, no other vaccines have been approved. For young children, it was found effective. However, it showed variable efficacy in adults. This variation was due to genetic variability among strains used to develop the BCG vaccine and media used to culture these strains as these criteria showed differences in immunogenicity. Moreover, prior exposure to non-tuberculous mycobacteria can affect the efficacy of BCG by inducing inhibition to its replication^28,29^. Many anti-tuberculosis vaccines have made their way to clinical trials and phase III trials to boost the effectiveness of the BCG vaccine^30^. However, it is time-consuming and expensive to develop and investigate proposed vaccines for efficacy and safety. Accordingly, immunoinformatics could be a gold approach to engineer vaccines and drugs compared to traditional in vitro and in vivo approaches. Of the successful stories of using immunoinformatics tools are developing vaccines against *rickettsia*^31^ and *E. coli*^32^, and several ones in late phases of clinical trials for *B. anthracis, S. aureus, Salmonella, C. Albicans, S. canis*, and many others^31,32^. Since 1990, mRNA vaccines were used against HIV-1^33,34^, Zika^35^, rabies^36,37^, influenza virus^38^, and many others. They were found to be effective and safe. Of the drawbacks to their usage were their instability due to degradation via RNases that are ubiquitous and innate immunity that can immediately recognize these structures as foreign^39,40^. However, headway was achieved to mRNA therapeutics since then.

Several in vivo and in vitro studies showed an association between epigenetic modifications and *M. tuberculosis* during infection. This epigenome regulator role is used by the *M. tuberculosis* to survive inside the host. Accordingly, innovative strategies to target this role can be exploited to develop either therapeutics or vaccines against this pathogen. These epigenetic modifications affect the genes of the immune system. However, it also affects the “trained immunity” term^24^. This theory indicates the occurrence of memory of the innate immune cells; as a result, quick and robust immunity occurs in secondary exposure to the infection^25^. This profile can be detected among individuals and are heritable for changes among populations^41^.

The main goal to achieve using vaccines is to provide a long-lasting memory. Thus, it is crucial to activating both B and T cells to realize this aspect. Accordingly, the host will efficiently and promptly respond to the infectious agent once encountered in the future^42,43^. However, the success of any vaccine is the proper use of specific antigens that are named epitopes^44^. Therefore, it is critical to predicting the epitopes that can elicit both B and T cells to implement in the vaccine construct. The HTL epitopes need to produce cytokines such as IFN-*γ*, IL-4, and IL-10^45,46^. Once antigen-presenting cells display the epitope for HTLs while using MHC class II molecules, the HTLs excrete these chemokines that play critical roles against pathogens. Once pathogens get eliminated, all immune cells perish except the memory cells^47^. The B cells possess receptors that are considered membrane-bound immunoglobulins that recognize antigenic epitopes^48^. Subsequently, these cells internalize and process the epitopes to present them to T cells using MHC II. As a result, these features had recognized by the T-cell receptor (TCR) of HTLs^49^. Accordingly, B cells differentiate to plasma cells that secrete antibodies that neutralize invaders and memory cells^49^.

For the previously mentioned reasons to manage the global tuberculosis crisis, we have designed *in silico* a multi-epitope mRNA vaccine while using the proteins of *M. tuberculosis*. These proteins affect the host epigenome, especially the genes related to the immune system. Each protein was investigated solely for epitopes that could elicit humoral and cell-mediated immunity. The IEDB is an online database to predict HTL and CTL epitopes from various experimentally derived immune epitopes^50^. The ABCpred was used to predict the linear B-cell epitopes. This server is an online database that performs the prediction using artificial machine learning^51^. The efficacy and safety of the epitopes were screened by analyzing them to be antigenic, non-allergen, and non-toxic using related online servers^52–54^. Specific linkers had used to connect between epitopes. The TLR4 agonist (RpfE) was added to the N terminus of the sequence to boost immunity. Ultimately, an immune simulation of the effect of the vaccine had performed to validate that this construct can involve both humoral and cellular responses as investigated. Three injections of the vaccine had used. All investigated proteins were extracted from the *M. tuberculosis* strain (ATCC 25618 / H37Rv). However, the selected epitopes need to lie in conserved regions of the bacterial genome. Accordingly, an alignment to all variants was performed. The chosen epitopes for our vaccine construct have 65 corresponding MHC alleles. However, six selected epitopes were subjected to molecular docking. It is critical to vaccine design that the ligand and receptor are robustly engaged^55–57^. Accordingly, molecular docking was performed to the selected epitopes with their corresponding MHC alleles. This tool is crucial to predict the binding affinity and pose during spontaneous bond formation. The lower the energy, the more tightly the receptor is bound to its ligand^58^. Interactions exhibited between the epitope and the pocket of MHC are also studied. Moreover, the vaccine construct also addressed population coverage for both CTL and HTL epitopes. Only the individuals with a specific MHC allele that recognizes the epitope will respond to the vaccine as there are more than a thousand human MHC alleles in the world^59^. Hence, the IEDB population coverage tool was used to predict this coverage. It was found that this vaccine covers almost 99.38% of the population.

**Figure 9:**
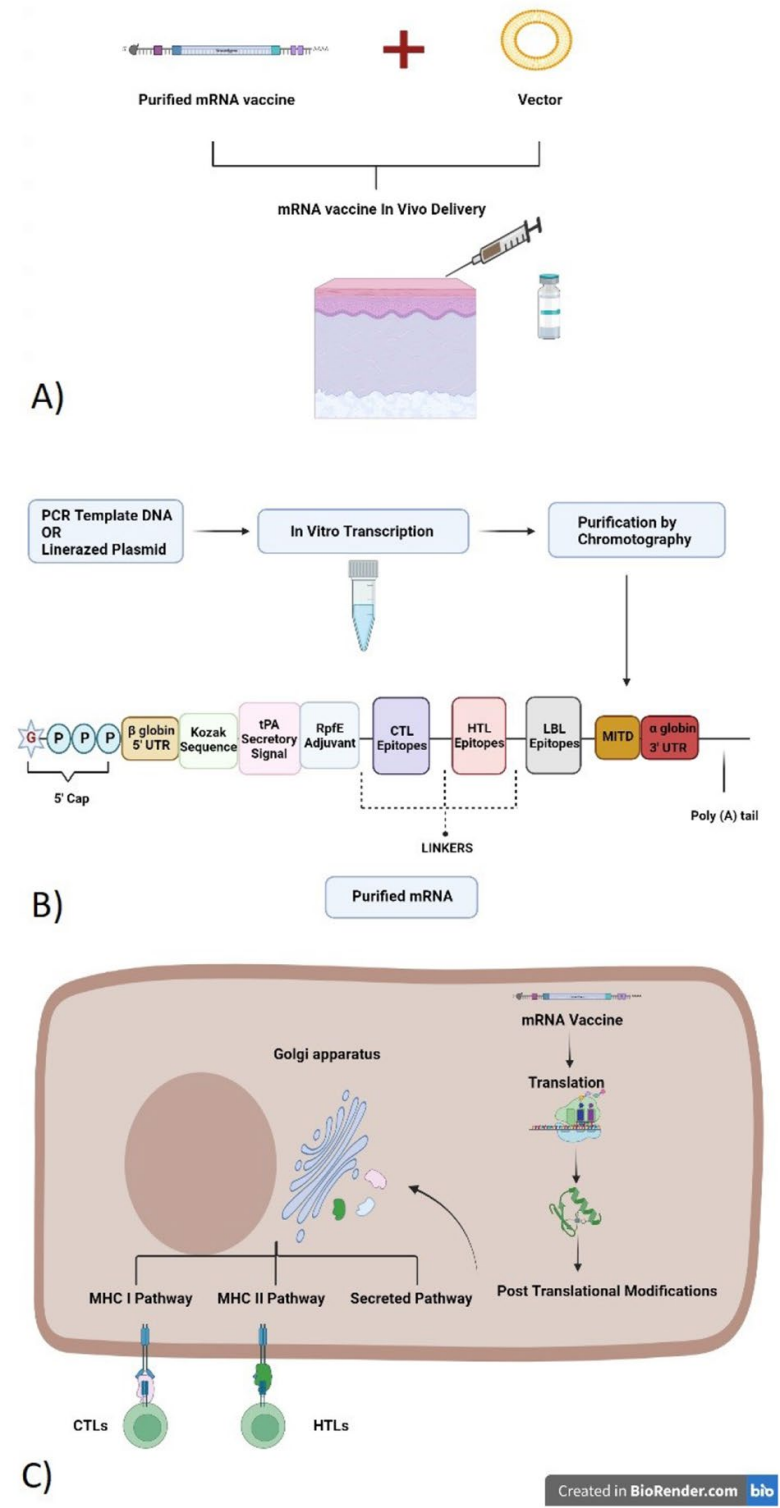
Proposed In vitro mechanism of production and In vivo method of delivery: (**A**) In vitro transcription of vaccine sequences (**B**) Vector-mediated delivery into the body and the mRNA transits to the cytosol (**C**) Mechanism of action of mRNA vaccine. Once it is translated into a protein in the cytosol, it undergoes PTMs and becomes a fully functional and properly folded protein. The tPA secretory signal and MITD sequences direct the peptides to specific compartments inside the cell (ER and Golgi apparatus) to either induce their secretion (LBL epitopes) or presentation (HTL and CTL epitopes) by the MHCI and MHCII.

Several structures were included in the mRNA vaccine to boost translation and stability capacity. Of these are: (1) 5′ m7G cap sequences^60^ (2) Poly (A) tail of length between 120-150 bps^61^ (3) Globin 5′ and 3′ UTRs that flank ORF of mRNA^62^ (4) The stop Codon^63^ and (5) the Kozak sequence^64^. Moreover, the tPA secretory signal sequence^65^ and MITD sequence^66^ to direct it to endoplasmic reticulum were added to improve the efficacy and allocation of the construct. Once it reaches the cytosol, the mRNA will enter the translation machinery to get post-translational modifications, producing an immune reaction (Fig 9). It is also essential to maintain the G: C content of the sequence for more stabilization and protein expression^67^. For administration purposes, mRNA can be encapsulated in a Lipid Nanoparticle (LNP) vector^35,38,68^. The route of administration is a factor to consider. The intramuscular and intradermal routes enhanced protein translation triple times than the intravenous route. Intranasal delivery can be an option that needs to be investigated on mice. The half-life of the vaccine is needed to be high, as more persistent antigens result in more immune response.

Adjuvants are used to boost immunity while considering both efficacy and safety measures without any risk of undesired activation and inflammatory response in some cases. Here, we used TLR4 agonist RpfE. It was found that mutation of TLR4 in mice results in towering bacterial load when infected with *M. tuberculosis*^69^. It is known that TLR4 is a receptor involved in recognition of *M. tuberculosis* and thus activates macrophages and dendritic cells that lead to activation of innate and adaptive immunity^70^. Further, the TLR-3 was docked with the vaccine to examine the capability of the constructed vaccine to bind with TLR on immune cells. The results revealed that the vaccine had a high binding affinity towards TLR-4 and TLR-3. Thus a probability of producing both innate and adaptive immunity. MD simulation was implemented to explore the complex’s stability, and the RMSD plot represented the steady binding of the complex. However, the naked mRNA vaccine can be used without an adjuvant^11^. This approach has both positives and drawbacks. It could reduce time and cost, exposing the sequence to be destroyed in vivo. Accordingly, a practical investigation is needed to determine if this step is needed.

As an appendix to the developing mRNA vaccine, it is essential to consider other factors such as its manufacturing and quality. Moreover, it is crucial to solve all related issues using the mRNA vaccine and ensure Good Manufacturing Practice (GMP). Thus, mRNA can be amplified in vitro using PCR or plasmid DNA with a site-specific RNA polymerase^71^. Then, purification of mRNA vaccine is performed as all contaminants of double-stranded RNA (dsRNA) are recognized and can induce Pathogen-associated Molecular Patterns (PAMPs) and type I interferon (Fig. 9). Thus, inhibition of translation and degradation of cellular mRNA could occur if this purification step is not performed^72^. The most successful method of purification is chromatography to obtain a purified mRNA of a specific length. This way resulted in robust translation to almost 1000-fold. Moreover, another method named PUREmessenger was used to produce purified mRNA. However, the best-reported method to produce an increase in protein production was when mRNA vaccine was both HPLC-purified and nucleoside modified^72^. For in vivo delivery purposes, a vector is used to deliver the mRNAs into the cytosol. The mRNAs enter the cellular translation machinery and post-translational modifications resulting in folded and fully functional proteins. The tPA secretory signal and MITD sequences direct the peptides to ER and Golgi apparatus for efficient secretion and presentation by MHC molecules (Fig. 9).

The IEDB database was used to predict the epitopes enlisted in the vaccine. This database contains spacious immune epitopes extracted from real experimental data^50^. The C-ImmSim webserver was used to profile the immunity of our designed vaccine. It uses specifically Position-Specific Scoring Metrix (PSSM) to simulate immune reactions. It possesses a collection of 6533 epitopes and 33 sets of human HLA alleles^73^. Essentially, the peptide sequence of the proposed mRNA vaccine was found to be stable, antigenic, non-allergenic, thermostable, and hydrophilic using immunoinformatics tools. It managed to induce an immune response once administered *in silico* with three injections. It was indicated that it could produce a memory after its exposure—high levels of B and T cells and the production of high levels of IFN-*γ* and IL-2. IFN-*γ* indicates cell-mediated immunity, and this chemokine can support B-cell proliferation, Ig isotype switching, and humoral response. Moreover, the activity of dendritic cells and macrophages and the Simson index indicate the generation of memory. Thus it can be concluded that this construct can be an excellent candidate to be considered a vaccine against tuberculosis.

In conclusion, the proposed construct shows desirable physicochemical properties and immunological responses. The performed immune simulation displayed that the vaccine elicited an immune response consistent with our hypothesis. Therefore, it is suggested to thrust forward in using this construct as a potential candidate for in vitro and in vivo studies against *M. tuberculosis* while using several serological assays to confirm the trigger of response in demand.

## Methods

**Figure 10:**
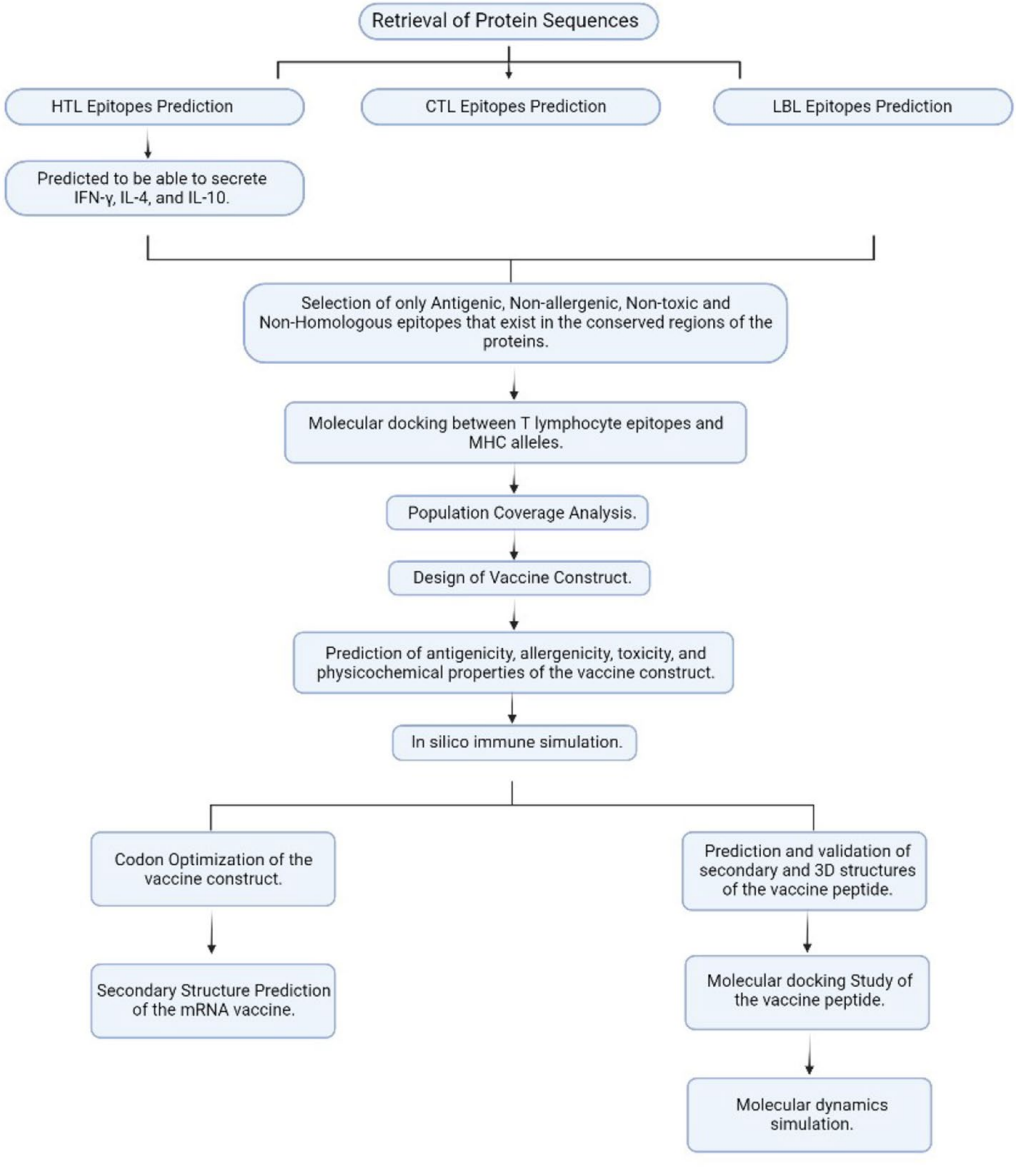
Workflow of RABA_MARZ_14.5.9 mRNA vaccine Development.

The Pipeline of our research methodology was outlined in Fig. 10.

### Retrieval of bacterial protein sequences

The Uniprot Knowledgebase at (http://www.Uniprot.org) was used to retrieve the amino acid sequences of (1) Histone Acetyltransferase Rv3423.1 (accession number-P9WKY5), (2) Rv2966c (accession number-I6XFS7), (3) EIS (Rv2416c accession number-P9WFK7), (4) esxL (Rv1198 accession number-P9WNJ5), (5) cobB (Rv1151c accession number-P9WGG3), (6) esxA (Rv3875 accession number-P9WNK7), (7) Rv3263 (accession number-P96868), (8) spoU (Rv3366 accession numberO50394) and (9) erm(37) (Rv1988 accession number-Q10838) in FASTA format. Moreover, the TLR4 agonist RpfE (Rv2450c, accession number-CCP45243.1) was retrieved to be used as an adjuvant in Fasta format^74^. The workflow of this research is outlined in Fig. 10.

### Immunoinformatics Analysis

#### B-cell epitopes prediction

For the prediction of linear B-cell epitopes, ABCpred, an online server (https://webs.iiitd.edu.in/raghava/ abcpred/ABC_submission.html), was used^51^. The ABCpred predicts linear B-cell epitopes using artificial machine learning. Each chosen protein was submitted individually with a 0.51 threshold. The length of epitopes was selected as 16mer. An overlapping filter was kept on. The top five epitopes of the results were studied further.

#### HTL epitopes prediction

Specifically, the MHCII server of Immune Epitope Database and Analysis Resources (http://tools.iedb.org/mhcii/) was used to predict HTL epitopes^50^. Independently, the fasta format of the amino acid sequences of the included proteins in this study was submitted to the server. NN-align 2.3 (Net MHC II 2.3) was used to predict the epitopes. The full HLA human reference set was utilized. The epitope length was specified to be 15mer. Finally, the results were sorted according to their adjusted ranks. Moreover, INFepitope (http://crdd.osdd.net/raghava/ifnepitope/predict.php)^75^, IL4pred (https://webs.iiitd.edu.in/raghava/il4pred/predict.php)^76^, and IL-10pred (https://webs.iiitd.edu.in/raghava/il10pred/predict3.php)^77^ servers were used to predict if these predicted epitopes can secrete IFN-*γ*, IL-4, and IL-10 respectively. All chosen epitopes showed the ability to secrete these cytokines.

#### CTL epitopes prediction

In the case of predicting CTL epitopes, the IEDB MHC I server (http://tools.iedb.org/mhci/) was use^50^. The selected proteins were submitted in fasta format. The ANN 4.0 setting was used to predict 9mer and 10mer epitopes. The complete human HLA reference set was used. Ultimately, the resulting peptides were sorted according to predicted IC50. Only epitopes with an IC50 over 500 were chosen.

### Human homology

All predicted peptides were searched using the Blastp algorithm (https://blast.ncbi.nlm.nih.gov/Blast.cgi?PAGE=Proteins) against the *Homo sapiens* (Taxid:9606) protein database to avoid the possibility of any autoimmunity. All peptides were selected further to be considered as possible non-homologous peptides in the vaccine if they have an E value higher than 0.05^78^.

### Prediction of epitope’s antigenicity, allergenicity, and toxicity

All selected epitopes were tested for their antigenicity, allergenicity, and toxicity. Antigenicity prediction utilized the VaxiJen web server (http://www.ddg-pharmfac.net/Vaxijen/VaxiJen/VaxiJen.html). The prediction is based on the physicochemical properties of epitopes in an alignment-independent fashion. Bacteria and a threshold of 0.4 were singled out^52^. To predict the allergenicity of epitopes, the AllerTop V.2.0 webserver (http://www.ddg-pharmfac.net/ AllerTOP) was used^53^. All parameters were kept to default. Ultimately, the ToxinPred server (https://webs.iiitd. edu.in/raghava/toxinpred/multi_submit.php) was used to predict and measure the toxicity of epitopes by generating all potential mutants while keeping all parameters to default^54^. For further steps in the research, only the epitopes that were found antigenic, non-toxic and non-allergenic were kept.

### Multiple sequence alignment

The NCBI database has been used to obtain all the variants of the selected proteins. The Bioedit 7.2 sequence alignment and analysis program was used to perform the alignment and viewing^79^. Then, we searched if all previously predicted epitopes lie in the proteins’ conserved regions.

### Molecular docking between T lymphocytes epitopes and MHC alleles

Some of the extracted T lymphocyte epitopes were evaluated for binding affinity for their corresponding MHC alleles while employing molecular docking simulation. The 3D structures of MHC alleles were downloaded from the RCSB PDB database. Then, PyMOL software was used to process the structures and remove unnecessary ligands^80^. Subsequently, the Swiss-PDB Viewer was used for energy minimization for the structures^81^. In parallel, all selected epitopes for docking were folded into three-dimensional using PEP-FOLD 3.5 server^81^, and energy minimized using the Swiss-PdBViewer. The ClusPro 2.0 server (https://cluspro.bu.edu/login.php) was used to dock each epitope with its corresponding MHC allele and calculate the binding affinity^82–84^. The pose and interactions were studied using PyMOL^80^ and Discovery Studio^85^, respectively.

### Population coverage analysis

The combined population coverage was measured for the selected T lymphocytes epitopes in the vaccine construct and their corresponding MHCI and MHCII alleles using the Population Coverage tool (http://tools.iedb.org/population/) in the IEDB database^59^. This measured value depends on the coverage of the MHC alleles that the epitopes in the construct recognize. It is because of the diversity in the distribution of MHC alleles against different geographical and ethnicity around the globe.

### Design of vaccine construct

The mRNA vaccine construct was proposed from the N- to C-terminus as the following: 5′ m7GCap-5′ UTR-Kozak sequence-tPA (Signal peptide)-EAAAK Linker-RpfE (Adjuvant)-GPGPG linker-HTL Epitopes-KK-LBL Epitopes-AAY Linker-CTL Epitopes-MITD sequence-Stop codon-3′ UTR-Poly (A) tail.

All proposed epitopes were linked through three linkers: AAY, KK, and GPGPG linkers^86^. These linkers exist to separate domains to let them act separately. They are cleavable, flexible, and rigid. An adjuvant Resuscitation-promoting factor (RpfE) (Rv2450c) was used to boost the adaptive immune reaction. In the mRNA vaccine, it is necessary to add a Kozak sequence which also includes a start codon^64^ in the ORF and a stop codon^63^. Moreover, two structures were added to the construct, which includes (1) The tissue Plasminogen Activator (tPA) secretory signal sequence (UniProt ID: P00750) in the 5′ region of the construct. This element is a signal sequence to help the secretion of epitopes once translated out of the cell if required^65^. (2) The MHC I-targeting domain (MITD) (UniProt ID: Q8WV92) in the 3′ locus of the mRNA vaccine. This sequence is needed to steer CTL epitopes toward the MHC-I compartment of the endoplasmic reticulum^66^. Moreover, it is crucial for stability purposes to add 5′ cap, 120–150 bases of poly(A) tail^87^, and *β* globin 5′ and *α* globin 3′ Untranslated regions (UTRs) to mRNA vaccines^61^. The vaccine construct was named RABA_MARZ_14.5.9.

### Prediction of antigenicity, allergenicity, toxicity, and physicochemical properties of the vaccine construct

The VaxiJen 2.0^52^ and ANTIGENpro servers^88^ were used to predict the antigenicity of the vaccine construct. This prediction is crucial as the antigenicity of an antigen can elicit immune response and form memory cells. In which, VaxiJen 2.0 performs predictions based on several physicochemical properties of the vaccine, while the ANTIGENpro server (http://scratch.Proteomics.ics.uci.edu/) is based on data collected from microarray analysis using machine learning algorithms. The input of the constructed mRNA vaccine includes only the amino acid sequence of a translated form of the ORF while excluding tPA and MITD sequences. Allergenicity of the construct was tested using AllerTOP 2.0 server^53^, while the toxicity of the vaccine was predicted using ToxinPred server^54^. Ultimately, the online webserver ProtParam (https://web.expasy.org/protparam/) was used to predict various physicochemical properties of the vaccine. These characteristics include the composition of amino acid, molecular weight, theoretical Isoelectric point (pI), Instability Index (II), Aliphatic Index (AI), and Grand Average of Hydropathicity (GRAVY)^89^.

### In silico immune simulation

The C-ImmSim, an online simulation server (http://150.146.2.1/C-IMMSIM/index.php), was used to perform a dynamic simulation of immune response for the vaccine construct while setting all criteria to default^73^. Its mode of action is based on epitopes in conjunction with lymphocyte receptors and hence simulates the immune response. Giving 2-3 doses within four weeks is recommended for most current vaccines. Hence, we used three doses of 1000 vaccine units over four weeks in the immune simulation of this study^90^. We set all parameters to default and three injections at time-step 1, 84, and 168, respectively.

### Codon Optimization of the vaccine construct

The peptide vaccine construct needs to undergo a codon optimization for efficient expression within the human cells. Accordingly, we used the GenSmart Codon Optimization Tool (http://www.genscript.com/) by GenScript (GS). Quality assessment of the optimized sequence was performed using the Rare Codon Analysis tools (http://www.genscript.com/) by GenScript (GS). The efficiency of translation of the mRNA is expressed as Codon Adaptation Index (CAI). The existence of any tandem unusual codons is indicated as Codon Frequency Distribution (CFD).

### Secondary Structure Prediction of the mRNA vaccine

The secondary structure of the mRNA vaccine was projected using the RNAfold tool (http://rna.tbi.univie.ac.at/cgi-bin/RNAWebSuite/RNAfold.cgi) of ViennaRNA Package 2.0. It uses McCaskill’s algorithm to compute the predicted secondary structure’s minimal free energy (MFE). This tool measured the minimal free energy (MFE) structure and the centroid secondary structure and their minimum free energy.

### Prediction and validation of secondary and 3D structures of the vaccine peptide

Excluding the tPA signal and MITD sequences, the PSIPRED server (http://bioinf.cs.ucl.ac.uk/psipred/) was used to predict the peptide’s secondary structure based on position-specific scoring matrices with an accuracy of 84.2%. The Robetta server (https://robetta.bakerlab.org/) was used to predict five possible three-dimensional structures of a peptide sequence^91^. The ProSA-web (https://prosa.services.came.sbg.ac.at/prosa.php), PROCHECK, and ERRAT (https://saves.mbi.ucla.edu/) were used to confirm the best structure.

### Prediction of conformational B-cell epitopes

The tertiary structure of the protein can induce new conformational B-cell epitopes. ElliPro, an online server (http://tools.iedb.org/ellipro/), has been used to predict the discontinuous B-cell epitopes in the protein structure^92^. Ellipro uses the geometrical characteristics of the 3D model. In comparison with other available prediction tools of discontinuous B-cell epitopes, ElliPro provides the highest AUC value of 0.732 for any protein model^93^.

### Molecular docking study of the vaccine peptide

The 3D structure of the vaccine peptide and toll-like receptor 4 (TLR-4) (PDB ID: 3FXI) or toll-like receptor 3 (TLR-3) (PDB ID: 1ZIW) were docked using the ClusPro server using the PIPER docking algorithm^84^. As a control, the RpfE adjuvant was also docked against TLR4 and TLR3. This server can generate different models based on different scoring schemes. The binding free energy (∆G), dissociation constant (Kd), and the percentages of charged and polar amino acids found on the non-interacting surface of the receptor-ligand 3D interaction were calculated using the PRODIGY tool of the HADDOCK server(https://haddock.science.uu.nl/)^94^. The PDBsum webserver was used to analyze and visualize the interactions between the receptor and ligand^95^.

### Molecular dynamics simulation

We performed dynamics simulation analysis for the TLR4-vaccine and TLR3-vaccine complex structures with the lowest binding energy using the iMODS server (http://imods.Chaconlab.org/); in order to confirm the physical motions of the atoms and molecules of the vaccine and its stability^96^.
